# 2D Metal-Organic Frameworks: Properties, Synthesis, and Applications in Electrochemical and Optical Biosensors

**DOI:** 10.3390/bios13010123

**Published:** 2023-01-11

**Authors:** Anamika Ghosh, Sana Fathima Thanutty Kallungal, Sundara Ramaprabhu

**Affiliations:** Alternative Energy and Nanotechnology Laboratory, Department of Physics, Indian Institute of Technology Madras, Chennai 600036, India

**Keywords:** two-dimensional, MOF, electrochemical, optical, sensor

## Abstract

Two-dimensional (2D) nanomaterials like graphene, layered double hydroxides, etc., have received increasing attention owing to their unique properties imparted by their 2D structure. The newest member in this family is based on metal-organic frameworks (MOFs), which have been long known for their exceptional physicochemical properties—high surface area, tunable pore size, catalytic properties, etc., to list a few. 2D MOFs are promising materials for various applications as they combine the exciting properties of 2D materials and MOFs. Recently, they have been extensively used in biosensors by virtue of their enormous surface area and abundant, accessible active sites. In this review, we provide a synopsis of the recent progress in the field of 2D MOFs for sensor applications. Initially, the properties and synthesis techniques of 2D MOFs are briefly outlined with examples. Further, electrochemical and optical biosensors based on 2D MOFs are summarized, and the associated challenges are outlined.

## 1. Introduction

Two-dimensional (2D) nanomaterials have sparked great interest among researchers owing to the interesting properties arising from their inherent layered, sheet-like morphology and nanometer regime thickness. The two-dimensionality imparts fascinating features such as mechanical flexibility, interesting charge transfer properties, huge surface areas, optical transparency, and plenty of accessible active sites to them, which finds applications in fields like flexible electronics, catalysis, sensors, gas separation, etc. [1]. Several 2D nanomaterials have been explored to date, the well-known ones being graphene and graphene oxide [2], and others such as metal oxides [3], layered double hydroxides [4], transition metal dichalcogenides [5], borophene [6], graphitic carbon nitride [7], etc. A recent addition to this family is the two-dimensional counterparts of metal-organic frameworks (MOF), which possess unique features and functionalities [8]. Similar to other 2D materials, they possess a huge specific surface area and excellent mechanical properties. Although the electrical conductivity of these structures is low as compared to other 2D materials such as graphene, the easy functional ability, and tunable properties make them interesting materials for diverse applications. A brief overview of some of the properties of 2D MOFs and other 2D nanomaterials are compared and summarized in Table 1 for better understanding.

**Figure 1:**
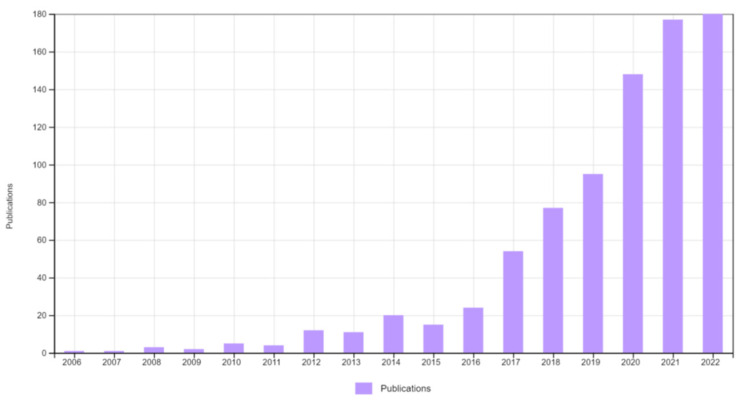
Graph depicting the number of publications on 2D MOFs over the years. Citation Report Graphic is derived from Clarivate *Web of Science*, Copyright Clarivate 2022. All rights reserved. (Keywords: 2D two-dimensional metal-organic frameworks MOF).

Metal-organic frameworks (MOFs) are porous, crystalline materials comprising metal-based nodes (ions or clusters) bonded to organic linkers via coordination bonds. They possess unique physical and chemical properties, such as high surface area, tunable porosity, fascinating morphologies, catalytic property, etc. [11]. They are of immense interest owing to the fact that these properties can be tuned based on the choice of metal or organic moiety. For instance, the pore size of MOFs can be altered from micropores to mesopores based on the length of the organic linkers used. Deng et al. [12] demonstrated isoreticular MOF-74 structures with different pore apertures (1.4–9.8 nm) obtained by combining short and long linkers. A more general strategy was developed by Yuan et al. [13] where a method termed linker labilization was used to obtain hierarchical porous structures. Likewise, MOFs provide the unique opportunity to rationally design and tune the properties by modulating the ligands and nodes. Due to these attractive features, MOFs have been widely explored for applications in gas storage [14], sensing [15], catalysis [16], energy storage [17], and much more [18]. The properties and applications of these MOFs are primarily determined by their structure, composition, size, and morphology [19]. A plethora of reports focusing on bulk MOF crystals and 1D MOF materials have been published over the years. However, it was only recently that there had been a surge of research related to 2D MOFs for various applications (Figure 1), as these materials couple the properties of both MOFs and 2D materials [20]. Compared to other materials such as graphene, carbon nanotubes (CNT), metal oxide, and metal particles, 2D MOFs exhibit several exciting properties, suitable for sensing applications. A few of such properties are listed below:

1. Tunable functionalities with varying ligands help to modulate 2D MOF and analyte molecule interaction through different interaction mechanisms such as hydrogen bonding, π-π stacking, electrostatic interactions, etc.
2. High surface area and lateral dimension provide better attachment of the molecules on the 2D MOF surface, leading to higher loading of probe molecules.
3. Adjustable pores for the targeted analyte through tunable building blocks that enable the high selectivity of 2D MOF.
4. Exposed metal sites on the surface of the 2D MOF accelerate the surface catalytic reaction.
5. Exposed metal sites on the surface provide excellent quenching properties, making them suitable for optical nucleic acid (NA) and immunosensor.

A comparison of 2D MOF-based sensors with other reported materials for the most common analyte glucose is summarized in Table 2. This review provides insight into the different 2D MOFs that have been employed in biosensors. Initially, their unique properties, synthesis strategies, and challenges involved are elaborated. Further, specific examples of 2D MOF-based electrochemical and optical biosensors are examined to understand and appreciate the use of these exciting materials for sensing applications. Finally, future perspectives and opportunities related to these materials are briefly discussed.

## 2. Unique Properties of 2D MOFs

Dimensionality has a strong influence on the properties of a material [29]. As a result, 2D MOFs possess drastically different properties as compared to 3D MOFs and are useful for applications where bulk MOFs are inadequate. For instance, 3D MOFs are limited by their poor electrical conductivity (due to their porosity, crystalline structure, and chemical composition) for applications in electrochemical sensors [30]. Nonetheless, a judicious combination of ligands and metal ions can yield conductive MOFs. Interestingly, the 2D counterparts of these MOFs demonstrate enhanced conductivity owing to the reduced charge transport path lengths [29]. One of the highest conductivity values reported (120 S cm^−1^) for 2D MOFs is by Low et al. [31] for a Cu-hydroxythiophenolate-based system. Another example is by Ohata et al. [32], where they observed a conductivity of 0.6 S cm^−1^ for nanosheets of nickel-hexaminotriphenylene (Ni-HITP) MOF. This was the highest observed for HITP-based MOFs with thicknesses less than 100 nm. The conductivities of the bulk and thin film analogs of this MOF were found to be 2 and 40 S cm^−1^ by Sheberla et al. [33].

2D MOFs also possess enormous surface area (due to the high aspect ratio) and abundant active sites, making them appealing for many applications, particularly sensing [20]. The high surface area provides a stable matrix for the efficient attachment of enzymes and promotes the adsorption of more analyte moieties, thereby increasing the sensitivity. The enhanced adsorption also improves the response time [34]. Additionally, the active sites on its surface are easily accessible owing to its 2D morphology. This enhances their catalytic properties by efficient utilization of these sites, and reduction of the diffusion barrier and mass transfer resistance [29,35,36]. In the case of optical sensors, the presence of more accessible sites facilitates the energy and charge transfer between the fluorophores and quenchers. Han et al. [37] obtained a lower detection limit for metal ions using nanosheets of a zinc-based MOF (ZSB-1) compared to its 3D form. He et al. [38] synthesized ultrathin (1.5 nm thickness) zirconium-based MOF nanosheets. These exhibited superior photocatalytic performance than their bulk counterparts.

Another attractive property of 2D MOFs is their excellent mechanical strength and flexibility, which finds applications in fields such as gas separation and sensor fabrication [39,40]. This is endowed by the presence of coordination bonds within the structure and the nanosheet morphology. Hermosa et al. [40] investigated the mechanical properties of a 2D CuMOF (flakes with the number of layers ranging from 1–50) and observed Young’s modulus of 5 GPa. Although this is lower than the typical value for 2D materials, this was enough to form an isolated, stable 2D structure of this MOF. 2D MOFs are also observed to be considerably more chemically stable than 3D MOFs. The chemical stability of these MOFs is essential for sensor applications where operation in aqueous environments with extreme pH values may be required. Recently, Zhang et al. [41] synthesized very thin (~3.97 nm) indium-porphyrin-based MOF (InTCPP) nanosheets with excellent stability in aqueous solutions with pH values ranging from 2–11. The nanosheets exhibited higher photocatalytic activity towards hydrogen production compared to 3D InTCPP. In a different report [42], bimetallic 2D MOF nanosheets (NiCoBDC) synthesized by the hydrothermal method were found to be stable in alkaline media. The material was soaked in 1 M KOH for 12 h to demonstrate its durability, after which the phase remained unchanged (as determined from X-ray diffraction).

**Figure 2:**
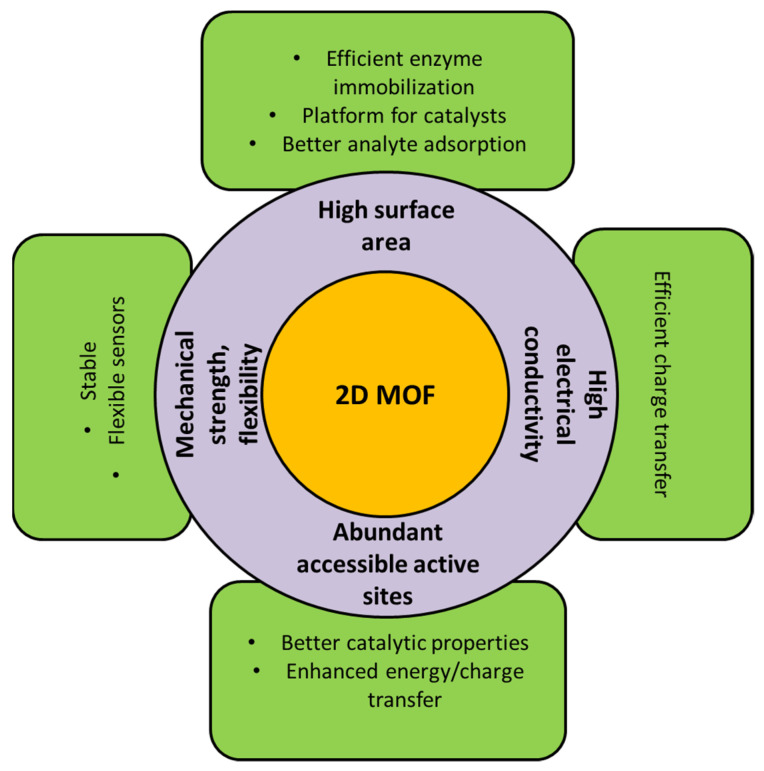
Diagram depicting the properties of 2D MOFs that makes it interesting for sensor applications.

Figure 2 summarizes the exciting properties of 2D MOFs which are appealing for sensor applications. All of the properties of 2D MOFs are predominantly dependent on the synthesis procedure, parameters such as solvent used, temperature, reaction time, etc. In the following section, some of the approaches to synthesizing robust, isolated two-dimensional MOF nanostructures are briefly discussed. 

## 3. Synthesis of 2D MOFs

**Figure 3:**
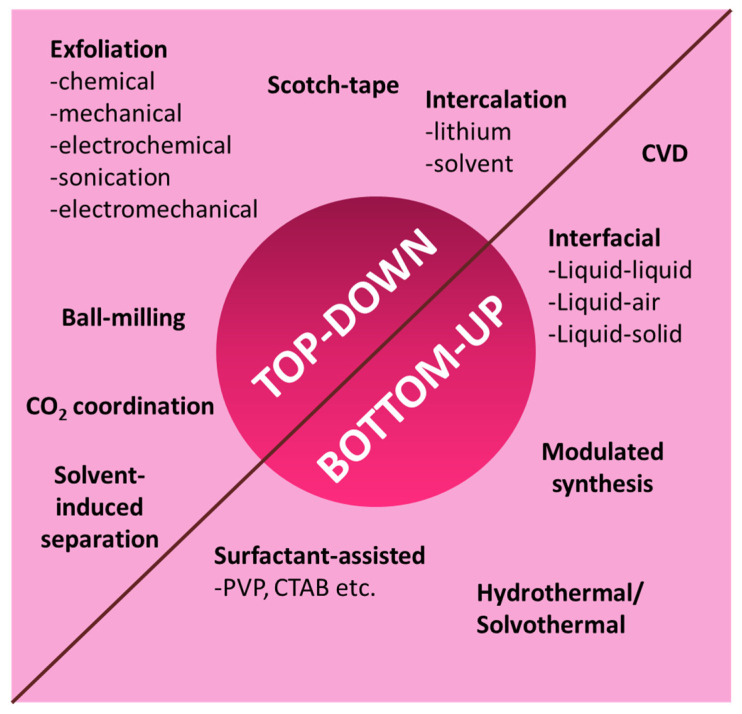
Schematic illustration of different synthesis routes of 2D MOF.

The fundamental challenge associated with 2D MOFs is synthesis, wherein it is necessary to strike a balance between yield, ease of synthesis, crystallinity, and stability. Several groups [39,43,44,45,46,47] have reported a variety of synthesis techniques for the formation of 2D MOFs. As with other 2D nanomaterials, the synthesis of 2D MOFs also follows one of the two main strategies—top-down or bottom-up approaches [8]. Figure 3 summarizes different synthesis routes for the preparation of 2D MOF.

### 3.1. Top-Down Synthesis

**Figure 4:**
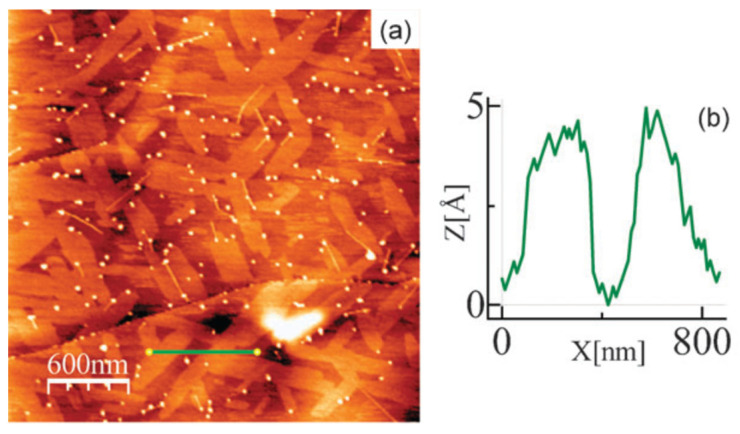
One-atom thick flakes of a Cu-based MOF obtained by top-down sonication method deposited on highly oriented pyrolytic graphite. (**a**) shows the topography image obtained using Atomic Force Microscope (AFM) and (**b**) depicts the height profile obtained along the green line in (**a**). Used with permission of [49]; permission conveyed through Copyright Clearance Center, Inc.

The top-down strategy is a simple yet powerful method that involves delaminating or stripping off layers (exfoliation) of the MOF from a bulk 3D MOF with a stacked-layer structure. The separation of the layers can be brought about by physical or chemical methods such as grinding [48], exfoliation (chemical [49], mechanical [50], electrochemical [51], electromechanical [52], or sonication [53]), intercalation (lithium, solvent) [54,55], etc., resulting in 2D MOF nanosheets with a single or few layers. The primary requirement here is that the MOF should be bonded by strong forces within the layers and weak forces (van der Waals, hydrogen bonding, π-π stacking, etc.) between the layers so as to enable easy separation of the layers [20]. The first report on the synthesis and characterization of a 2D MOF was by Amo-Ochoa et al. [49] (Figure 4). They isolated and studied single-atom-thick, bi-dimensional Cu_2_Br(IN)_2_ (IN—isonicotinato) synthesized by sonicating its 3D counterpart (obtained by hydrothermal reaction) in water. The sonication separated the layers by minimizing the interactions between them. Sonication treatment in other solvents such as acetone [56], ethanol [57], methanol [58], N,N-dimethylformamide [59], etc., or a mixture of two or more of these has also been reported [60]. The choice of solvent is a critical parameter and plays a crucial role in exfoliation. The solvents, in addition to separating the layers of the MOF, help stabilize the nanosheets by avoiding restacking. For instance, Tan et al. [61] reported the top-down synthesis of 2D M-DMS and M(2,2-DMS) MOFs (M = Mn, Co, Zn; DMS- dimethyl-succinate) by sonication in different solvents. Ethanol was identified to be the most effective for exfoliation and prevention of restacking. Through systematic studies, they also found that the formation of the 2D structure was primarily dependent on the bulky nature of the organic ligand rather than the cation. Taking a cue from this, another group [43] designed a MOF with bulky ligands (with 3-methoxypropoxy groups) in order to ease the exfoliation in different solvents. 

**Figure 5:**
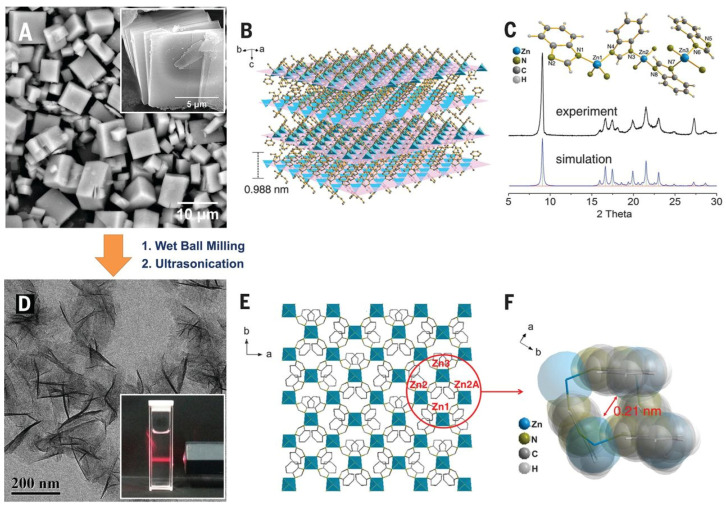
Top-down synthesis of Zn_2_(BIM)_4_ nanosheets from its corresponding crystals. The scanning electron microscopy (SEM) images of the crystals are shown in (**A**) with the layered structure clearly visible in the inset. The top-down treatment (wet ball milling + ultrasonication) exfoliates it into nanosheets as observed from the transmission electron microcopy (TEM) images (**D**). The inset in (**D**) shows the Tyndall effect exhibited by its colloidal suspension. The phase formation of the prepared MOFs is confirmed by X-ray diffraction (**C**). The schematic representation of the MOF architecture before and after the exfoliation treatment is shown in (**B**,**E**), respectively. (**F**) represents the space-filling model of a four-membered ring of the MOF nanosheet. From [60]. Reprinted with permission from AAAS.

Apart from sonication, other exfoliation techniques such as the Scotch-tape method [62] (mechanical, well-known for graphene synthesis), wet ball-milling [60], solvent-induced separation [63], etc., have also been reported. All of these methods suffer from the limitation of low exfoliation yield. Several groups have reported a combination of these strategies to improve the same. One such example is depicted in Figure 5. As mentioned earlier, these methods are only suitable for 3D MOFs with a layered structure. However, recently Zhou et al. [64] demonstrated the top-down synthesis of a 2D non-layered MOF by CO_2_-coordination. They proposed that simultaneously dissociating the ligand and coordinating a capping agent in a 3D non-layered MOF can prepare high-quality 2D MOFs. This necessitated the selection of a suitable microenvironment to carry out the defect generation and coordination. They proved this concept by treating 3D non-layered CuBTC (BTC—1,3,5-benzenetricarboxylate) in a solvent mixture containing supercritical CO_2_. This led to the dissociation of the BTC ligands, followed by the coordination of the CO_2_ molecules. The resulting Cu(II) dimer was square planar, bonded to two –COO^−^ groups of the ligand and two –HCOO^−^ groups of the CO_2_. These groups can attach to trigonal BTC molecules to form the non-layered 2D MOF structure.

Although these approaches are simple and robust, the yield obtained from these methods is too low for practical applications. The exfoliation rate is also low in most cases [8]. Additionally, for layered MOF structures, restacking the layers can threaten the stability of the MOFs, if not accounted for, owing to the weak forces involved. In this context, synthesis approaches different from the top-down strategy need to be explored.

### 3.2. Bottom-Up Synthesis

The bottom-up approach involves building the 2D structure from the metal ions and ligands by chemical reactions. This method allows more chemical flexibility and tenability [20,65]. For instance, for layered 2D MOFs, the number of layers in the MOF nanosheets can be controlled by tuning the reaction parameters. This is also the widely adopted strategy for non-layered 2D structures. The critical factor in the bottom-up method is suppressing the MOF growth in one direction without affecting the growth in the other two directions [39,66]. For layered-type MOFs, this implies preventing the layers from stacking up in the vertical direction. This can be easily done using interrupters (like surfactants) which can prevent the stacking during the formation process [20]. For non-layered MOFs, selective growth in two directions is challenging yet possible. Most of the strategies to achieve growth restriction involve synthesis in a confined 2D space, creating high surface energy structures, and chemically modifying the surfaces [67]. Based on these concepts, several methods have been reported for synthesizing 2D MOFs—interfacial synthesis, surfactant-assisted synthesis, modulated synthesis, hydro/solvothermal method, sonication synthesis, and so on.

**Figure 6:**
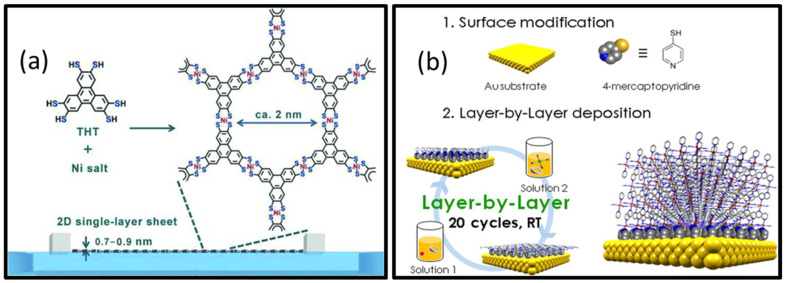

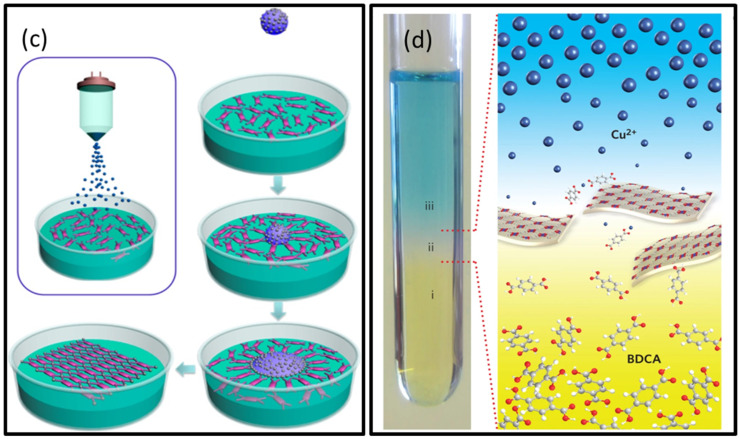
Schematic illustrations of different interfacial methods employed for the synthesis of 2D MOFs: (**a**) Formation of a single-layer sheet of Ni-based MOF at a liquid-air interface by Langmuir-Blodgett method. Reproduced from [69] Copyright 2015 Wiley Materials. (**b**) Synthesis of 2D Fe-MOF at a liquid-solid interface. Reprinted (adapted) with permission from [70]. Copyright 2017 American Chemical Society. (**c**) Formation of MOF nanosheets at a liquid-liquid interface by spraying technique. Reprinted (adapted) with permission from [68]. Copyright 2017 American Chemical Society. (**d**) Formation of ultrathin nanosheets of CuBDC MOF by a three-layer preparation approach. Reprinted by permission from [Springer Nature Customer Service Centre GmbH]: [Springer Nature] [Nature Materials] [Metal-organic framework nanosheets in polymer composite materials for gas separation, Tania Rodenas et al.] [Copyright 2014 Macmillan Publishers Limited] (2015) [39].

In the interfacial method, the synthesis reaction between the metal nodes and the ligands is confined to a 2D interface region, thereby forming nanosheets [67]. The interface can be liquid-liquid, liquid-air, or liquid-solid. The liquid-liquid [68] interface is formed by two immiscible solvents, one in which the metal is dissolved and another in which the ligand is dissolved. Typical examples of solvent mixtures used are water-ethyl acetate and water-dichloromethane. The formation of MOFs from their constituents occurs on the interface driven by diffusion. In the liquid-air interface method [69], a small quantity of organic solvent is added to a liquid, which evaporates to form the interface. By using a uniform monolayer of ligands, this interface allows the formation of ultra-thin, single-layer MOF nanosheets. Utilizing a Langmuir-Blodgett trough can control the nanosheet thickness as well as maximize the yield. The liquid-solid interface [70] involves layer-by-layer growth of the MOF on a chemically modified surface. Synthesis of MOF nanosheets at gas-solid interfaces has also been reported. However, it requires ultra-high vacuums and flat metal surfaces. Rodenas et al. [39] adopted a three-layer synthesis approach to synthesize copper-1,4-benzenedicarboxylate (CuBDC) MOF. The solutions allow acute control of the diffusion and kinetics of the reactants, thereby producing ultra-thin (5–25 nm) nanosheets with high yield. The thickness was tunable based on the solvent and reaction temperature. The schematic illustrations for different interfacial methods for synthesizing 2D MOFs are given in Figure 6.

**Figure 7:**
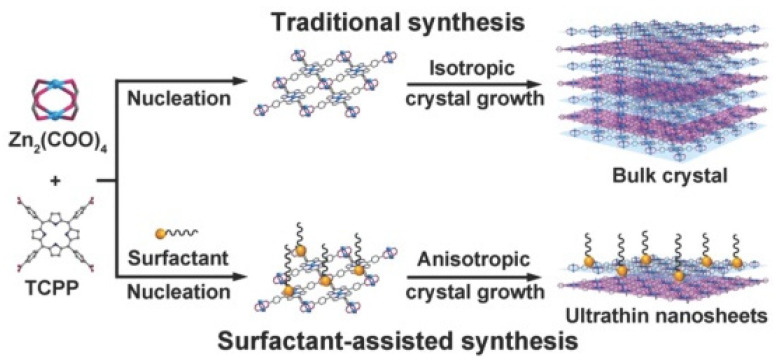
Scheme depicting the surfactant-mediated synthesis of 2D MOFs. The traditional synthesis method yields bulk crystals due to isotropic growth. The incorporation of the surfactant on the MOF surface results in anisotropic growth, thereby producing ultrathin nanosheets. Reproduced from [47]. Copyright 2015 Wiley Materials.

Another widely reported method is the surfactant-assisted synthesis [34,41,47]. The presence of a surfactant (like polyvinylpyrrolidone (PVP), cetyltrimethylammonium bromide (CTAB), etc.) lowers the surface energy of the as-prepared MOF nanosheets by preventing their aggregation. For instance, Zhao et al. [47] utilized PVP for the synthesis of 2D TCPP-based MOFs (Figure 7). The PVP restricted the MOF stacking as well as stabilized the synthesized nanosheets. Instead of surfactants, certain small molecules such as pyridine and acetic acid (termed modulators) have also been employed for 2D MOF synthesis (modulated synthesis) [71]. These molecules possess functional groups similar to that of the linkers, and hence compete to coordinate with the metal nodes. This competition regulates the growth of MOFs. The modulators selectively coordinate to specific planes of the MOFs, which block the growth along that direction. The preparation of non-layered MOFs has also been reported using this method [58].

Multiple other techniques, such as chemical vapor deposition [72], hydro- or solvothermal [73,74] synthesis, etc., have also been reported for the synthesis of MOF nanosheets. In most synthesis methods, there is always a competitive formation of 3D MOF occurring along with the 2D MOF. Oh et al. [75] found that the selective formation of 2D MOFs over their 3D counterparts critically depended on the relative ratio between the metal ions and organic ligands. They also demonstrated a modified bottom-up synthesis of 2D MOF layers by a combination of ultrasonication and surfactant-assisted methods.

Combining the top-down and bottom-up approaches for the synthesis of 2D MOFs is also becoming popular. Chaudhari et al. [76] synthesized nanosheets of a zinc-based MOF using a rapid self-assembly technique followed by sonication. Similarly, Junggeburth et al. [46] employed CTAB-assisted synthesis followed by exfoliation to prepare ~10 nm thick sheets of a zinc-based MOF. In summary, each synthesis method poses its own pros and cons. A judicious choice of the method and thorough optimization of the reaction parameters is necessary to synthesize 2D MOFs with desired properties and suitable for particular applications. Extensive reviews on the synthesis methods can be found elsewhere [20,29,65]. As mentioned in the previous section, 2D MOFs have been utilized in various applications owing to their unique properties. One of the extensively reported applications is in sensors, wherein they have been used for the detection of various analytes such as humidity [77], hydrogen [78], metal ions [37,53], explosives [79], pesticides [80], DNA [47,81], glucose [82,83], proteins [84,85], disease biomarkers [84,86], antibiotics [87,88], and much more. In the following sections, electrochemical and optical sensors employing 2D MOFs for the detection of biological analytes are discussed.

## 4. 2D MOFs for Electrochemical Biosensor Applications

Recently, MOFs have been widely explored for electrochemical biosensor applications. One of the attractive characteristics of MOF is its tunable pore size by changing the ligand type. Therefore, MOF acts as molecular sieves, improving selectivity towards a specific analyte. Additionally, the accessible pores of the MOF allow the diffusion of the analytes throughout the MOF skeleton, thus improving the catalytic activity of the MOF as an electrochemical biosensor. Often the bulk MOF suffers from poor dispersibility, increasing the possibility of detaching from the catalyst substrate during the sensing measurement. In that case, 2D MOF may overcome the challenges of 3D MOF. 2D MOFs are endowed with a high surface area that encourages the better adsorption of biomolecules. Besides, the exposed metal sites, porous network, enhanced mass transfer, and good dispersion make 2D MOF suitable for electrochemical biosensors. In the below section, we will discuss the recent advancements in the field of 2D-MOF-based electrochemical biosensor.

### 4.1. 2D MOF as a Nonenzymatic Sensor

The development of 2D MOF as an electrochemical sensor is drawing the attention of research scientists. Moreover, 2D MOFs have recently been probed as nonenzymatic sensors since they offer many advantages over enzymatic ones. Nonenzymatic sensors are cost-effective, stable over time and pH, easy to use, and have no storage issues. Due to their high surface area, ultrathin nanosheet nature, tunable porosity (which contributes toward selectivity), and enhanced electrocatalytic activity, 2D MOFs have the potential for electrochemical biosensing. 

**Figure 8:**
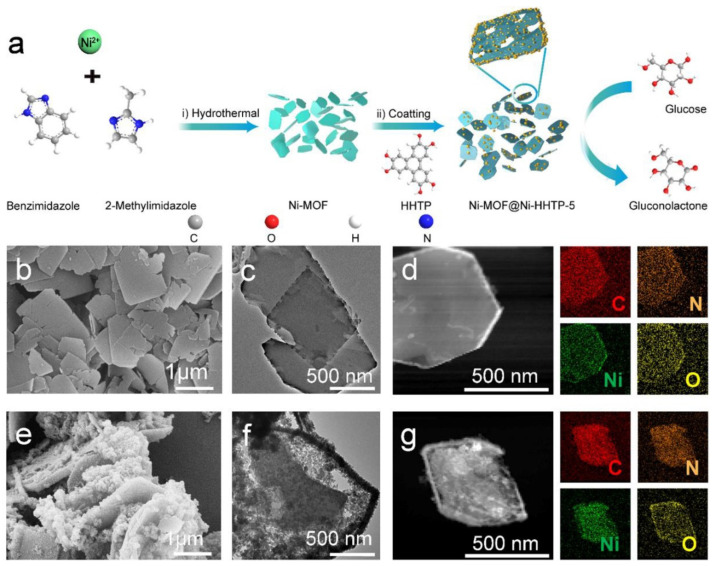
(**a**) Schematic representations of Ni-MOF@Ni-HTTP-5 synthesis and its glucose biosensing application, (**b**,**c**) SEM and TEM morphologies of Ni-MOF NS, (**e**,**f**) SEM and TEM images of Ni-MOF@Ni-HTTP-5, (**d**,**g**) STEM and elemental mapping images of Ni-MOF-NS and Ni-MOF@Ni-HTTP-5 NSs. Reprinted from [28], Copyright (2022), with permission from Elsevier.

Wang et al. tuned the coordination environment in 2D Ni-MOF by a π-conjugated molecule and synthesized MOF@MOF structures (Figure 8). They used conductive 2,3,6,7,10,11-hexahydroxytriphenylene (HHTP) as the organic linker [28]. HHTP acts as an extended π-conjugated molecule which can help in enhancing mobility and conductivity. In this work, the first Ni-MOF nanosheet was synthesized by hydrothermal method using nickel nitrate (Ni(NO_3_)_3_) as a precursor for the metal center and benzimidazole and 2-methylimidazole as organic ligands. In the next step, synthesized Ni-MOF and HHTP were dispersed in a mixture of N,N-dimethylformamide (DMF), and deionized (DI) water and kept at 85 °C for 6 h. Through the time-dependent studies, they observed that HHTP particles got attached mainly at the margins or periphery of the NiMOF sheet, and after 2 h, HHTP particles started etching the Ni MOF sheet. In brief, HHTP coordinates with the free Ni^+2^ of Ni-MOF and simultaneously etches the Ni-MOF nanosheets. Further, they studied the electrochemical glucose sensing properties of porous Ni-MOF@Ni-HHTP-5 (5 indicates 5 mg loading of HHTP) nanosheet in potassium hydroxide (KOH) (0.1 M) solution. At a constant potential of 0.6 V, Ni-MOF@Ni-HHTP-5 electrocatalyst could sense glucose concentrations within 0.5–2665.5 µM with a sensitivity of 2124.90 µA mM^−1^ cm^−2^ and LOD of 0.0485 µM. Here, although the glucose-sensing mechanism depends on the change of valence of the Ni metal center, the inclusion of HHTP in the NiMOF nanosheets encourages the better utilization of Ni^+2^ ions, accelerating the exposure of the metal sites that, in turn, enhances the conductivity and catalytic activity of the sensor. 

Stolz et al. studied the electrochemical properties of the different facets of conductive MOF and their application in sensing neurotransmitters such as dopamine (DA) [89]. They synthesized Ni_3_(HHTP)_2_ and Co_3_(HHTP)_3_ MOF in two different methods: (a) hydrothermal and (b) Interfacial synthesis. Morphology of the hydrothermally synthesized MOFs appeared rod-shaped and defined by {100} family of planes, indicating that terminal edge sites were exposed surface sites. On the other hand, MOF synthesized in interfacial synthesis revealed sheet-type morphology and was defined by {001}, where the dominant surface sites were a basal plane. They further examined the electrochemical performances of {001} and {100} planes of M_3_(HHTP)_2_ MOFs in different biological analytes such as DA, uric acid (UA), serotonin (5-HT), ascorbic acid (AA). They observed improved peak current (I_p_) for MOF over bare GCE in positively charged DA, 5-HT solution while the performance deteriorated compared to bare glassy carbon electrode (GCE) in case of solution with negative analytes such as UA, AA, etc. However, the I_p_ value drastically improved in the DA solution with {001} facet of M_3_(HHTP)_2_ MOFs compared to the {100} facet. The enhancement of peak current is attributed to probably two factors: (a) complementary Coulombic interactions between positively charged DA and MOF interface, (b) available and favorable adsorption of DA on {001} family of planes compared to {100} of MOF. Their integrated sensor Ni_3_(HHTP)_2_/GCE showed linearity of 0–6 µM in 0.1 M PBS and 0–2 µM in simulated cerebrospinal fluid with a LOD of 9.9 ± 2 nM and 214 ± 48 nM, respectively.

**Figure 9:**
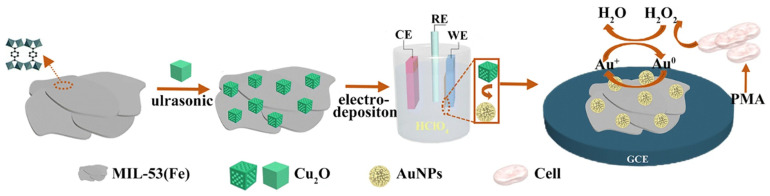
Schematic representation of synthesis of Cu_2_O mediated Ag/MIL-53(Fe) and real time monitoring of H_2_O_2_ from living cell A549. Reprinted by permission from [Springer Nature Customer Service Centre GmbH]:[Springer Nature] [Analytical and Bioanalytical Chemistry] [Cu2O-Mediated Assembly of Electrodeposition of Au Nanoparticles onto 2D Metal-Organic Framework Nanosheets for Real-Time Monitoring of Hydrogen Peroxide Released from Living Cells, Sha Chen et al.] [COPYRIGHT 2020] (2021) [90].

Chen et al. fabricated a nonenzymatic sensor of Cu_2_O-mediated growth of Au nanoparticles (NPs) on 2D MIL-53 (Fe) for real-time monitoring of hydrogen peroxide (H_2_O_2_) from living cells (Figure 9). In this work, they prepared Cu_2_O NPs and MIL-53(Fe) separately, and later, the composite of the two was prepared by sonicating in Nafion solution [90]. MIL-53 (Fe) was synthesized using iron chloride (FeCl_3_, 6H_2_O) as the metal source and terephthalic acid (H_2_BDC) as the organic linker. Hydrothermally synthesized MIL-53 (Fe) showed a 2D stacked sheet-type structure. Further, Au NPs were decorated on Cu_2_O/MIL-53(Fe)/GCE via electrodeposition method in HAuCl_4_ solution at −0.2 V. Au@Cu_2_O/MIL-53(Fe)/GCE sensor could sense H_2_O_2_ within a linear range of 0.01–1.52 mM with a sensitivity of 0.351 mA mM^−1^cm^−2^ and LOD of 1.01 µM. Sensing of H_2_O_2_ is related to the change of valence of Au (Au^0^ →Au^+1^); however, 2D MIL-53(Fe) here provides a high surface area and a matrix to anchor agglomeration free NPs. Additionally, pores of MIL-53(Fe) sheets also helped for selective detection of H_2_O_2_. Further, Au@Cu_2_O/MIL-53(Fe) was applied for real-time monitoring of released H_2_O_2_ from A549 lung cancer cells, and the sensor could detect an H_2_O_2_ concentration of 0.042 fM. 

Bottier et al. demonstrated a composite of 2D Co-MOF/Nafion, which shows an enzyme-like activity toward H_2_O_2_ sensing. Co-MOF was synthesized in a solvothermal approach using [(Co_4_O_4_)(OAc)_4_(py)_4_] as a building block [91]. H_2_O_2_ sensing performance was further evaluated in pH 7 using voltammetry and chronoamperometry techniques. Voltammetry anodic current vs. H_2_O_2_ concentrations plot matched well with the Michaelis-Menten reaction scheme, thus confirming the enzyme mimicking characteristics of the developed sensor. The enzyme mimicking characteristics is probably due to the specific coordination environment of the synthesized 2D MOF, where the metal centers are coordinated by a suitable organic ligand. Thus, the coordinated metal centers mimic the enzyme redox cofactor environment inside the MOF and exhibit ‘enzyme mimic’ characteristics. The sensor displayed linear ranges within 5 µM^−1^ mM and 1–10 mM with a corresponding sensitivity of 570 ± 5 A cm^−2^ mM^−1^ and 395 ± 10 A cm^−2^ mM^−1^. Periodically arranged Co metal centers with the optimum length organic ligand in the Co-MOF sheet allow fast diffusion of the electrolyte where exposed metal centers accelerate the redox reaction for H_2_O_2_ detection. 

Lu et al. designed a heterostructure based on 2D/2D NiCo-MOF/Ti_3_C_2_ for nonenzymatic detection of acetaminophen (AP), DA, and UA. In this heterostructure, the lamellar Ni-Co MOF sheet contains abundant unsaturated metal sites that contribute to the catalytic activity [92]. Ti_3_C_2_ offers hydrophilicity and conductive matrix support for the agglomeration-free dispersion of NiCo-MOF sheets. The developed sensor can detect AP (working potential 0.346 V vs. SCE), DA (working potential 0.138 V vs. SCE), and UA (working potential 0.266 V vs. SCE) simultaneously in a linear range of 0.01–400 µM, 0.01–300 µM and 0.01–350 µM with a detection limit of 8, 4, and 6 nM, respectively in 0.1 M phosphate buffer solution (PBS pH 7.4). They attributed the electrocatalytic activity of NiCo-MOF/Ti_3_C_2_ heterostructure to three factors: (a) metal-O-metal chains create a conductive pathway through the MOF lamellar sheet, (b) exposed unsaturated Ni and Co sites that serve as a catalytic center for AP, UA, DA detection, (c) two-dimensionality of Ni-Co MOF accelerates the electron hopping across the metal centers and also boosts the electrolyte diffusion through the pores of the MOF, and (d) finally Ti_3_C_2_ provides a 2D conductive matrix to prevent accumulation of Ni-Co MOF nanosheets, thus enhancing the exposure of more catalytic sites. The proposed sensor could detect AP, UA, and DA from serum and urine samples with a 98.1–102.2% recovery rate.

Wang et al. developed a facile approach to design a 2D array of MOF-based flexible electrode for sweat biosensor via interfacial synthesis and dip coating method [93]. In brief, first, they synthesized amine group functionalized (-NH_2_) graphene paper (NH_2_-GOP). Then Cu_3_(btc)_2_ MOF (btc-1,3,5-benzenetricarboxylic acid) was prepared through the interfacial emulsion process and later transferred on the NH_2_-GOP through the dip coating method. This leads to the 2D assembly of Cu_3_(btc)_2_ nanocubes on the freestanding graphene paper. The developed NH_2_-GO-Cu_3_(btc)_2_ flexible electrode was further tested to monitor glucose and lactate in sweat. In such MOF-based electrodes, electrochemical sensing is mainly ascribed to the extended conductive network through the MOF skeleton since electron hopping is allowed in the adjacent metal centers of the MOF. The sensor showed linearity for lactate concentrations of 0.05 mM–22.6 mM with a sensitivity of 29 µA cm^−2^ mM^−1^ and for glucose concentrations of 0.05–1775.5 µM with a sensitivity of 5.36 mA cm^−2^ mM^−1^. Their fabricated sensor also suffers minimum interference from other bioanalytes and has shown promising performance when examined for flexibility and dynamic test.

In another work, Wang et al. developed 2D Ni, Co, and NiCo-based MOF using 1,4- benzene dicarboxylic acid (BDC) as the organic ligand [94]. Synthesized MOFs showed lamellar sheet-type morphology with smooth surfaces. Further, they implemented the synthesized MOFs for urea detection. Urea is a human metabolite, and the content of urea in the blood is an essential indicator of kidney or liver functions. No voltammetric response of Co-MOF with urea addition was observed. Although NiCo MOF and Ni-MOF showed enhanced current response with the addition of urea, the urea sensing response for Ni MOF was more pronounced. This is attributed to the ultra-thin thickness and high surface area of Ni-MOF nanosheets. Ni-MOF nanosheets showed chronoamperometric linear response for urea concentrations of 0.5–832.5 µM with an excellent sensitivity of 1960 µA mM^−1^ cm^−2^ in 0.1 M KOH solutions at operating potential 0.45 V.

### 4.2. Nucleic Acid-Based Sensor

**Figure 10:**
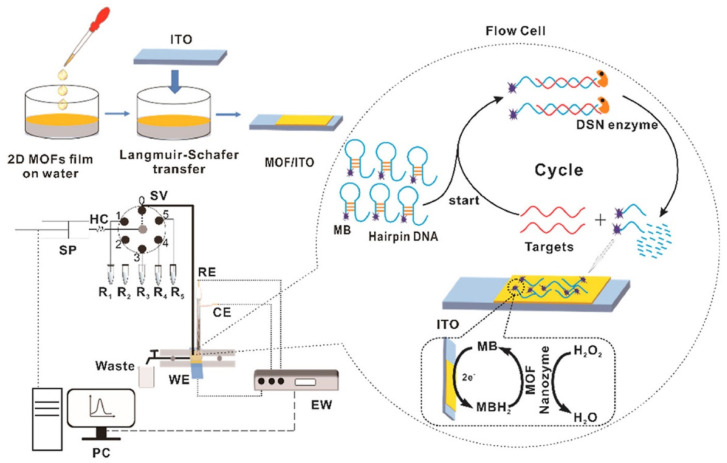
Schematic of homogeneous flow assay for microRNA detection, SP-syringe pump, SV-six port multiposition valve, HC-holding coil, WE-working electrode, RE-reference electrode, CE-counter electrode, EW-electrochemical workstation, MB-Methylene Blue. Reprinted from [95], with permission from Elsevier.

Nucleic acid-based sensors provide massive opportunities for the sensitive and rapid detection of several proteins and biomarkers. As previously discussed, 2D MOF offers a high surface area for the adsorption of biomolecules and sometimes shows peroxidase mimic character; they can be utilized for the nucleic acid-based electrochemical sensor. Apart from these, 2D MOFs possess several other advantages. 2D MOFs allow tight binding with the probe molecule through π-π stacking. Further, they offer a high loading capacity of probe molecules, open metal sites, and tunable organic functionalities that contribute to excellent quenching properties and boost sensing performance. Additionally, the fluorescence quenching efficiency can be effectively improved by modulating the structure of MOF, varying the metal center and ligand, and regulating the ratio of center metal ions and organic ligands. Further, the 2D MOF-based electrochemical NA sensor has also drawn attention owing to the robustness, simplicity, high sensitivity, and rapid detection characteristics of the electrochemical sensing mechanism. In the following section, we will discuss the 2D MOF-based electrochemical NA sensor. Wang et al. designed a flow homogeneous assay based on Co-MOF-based 2D nanozyme for successive microRNA assay [95] (Figure 10). Co-MOF nanosheet was synthesized in an ultrasonication method using Co^+2^ as metal nodes and H_2_BDC as an organic ligand. Transmission electron microscopy (TEM) morphology revealed the lateral size of the synthesized nanosheet was about 100–300 nm, while the thickness of the nanosheets below 1 nm was confirmed through (Atomic force microscopy) AFM. The working electrode was prepared by transferring the 2D Co-MOF sheet to the (Indium tin oxide) ITO electrode via the Langmuir-Schafer approach. They further developed a flow homogeneous microRNA assay that doesn’t require DNA recognition elements or functionalization of the working electrode. MOF/ITO electrode could differentiate ssDNA from double strands DNA (dsDNA) due to the π-π stacking. In the flow homogeneous microRNA assay, they used duplex specific nuclease (DSN) enzyme. First, a methylene blue (MB) labeled hairpin DNA (HP) was kept and gave a negligible electrochemical response. In the presence of the target, i.e., microRNA, microRNA hybridized with HP DNA to form an RNA/DNA hybrid system. Further, DNA from the DNA/RNA hybrid system gets hydrolyzed by the DSN enzyme and releases microRNA and short DNA in the solution. MicroRNA can hybridize with HP DNA again, whereas MB-labeled short DNA showed a strong affinity and diffusivity towards 2D MOF/ITO electrode. In the presence of H_2_O_2_, oxidized MB gets electrochemically reduced at the MOF/ITO electrode, leading to a distinct enhancement in the electrochemical signal. Thus, based on the above mechanism, the presence of microRNA in repeated cycles yields a large amount of MB labelled short DNA, which amplifies the electrochemical signal. Additionally, the chemical and enzyme-like activity of 2D MOF nanozyme also helped to boost the electrochemical signal. The microRNA assay flow homogeneous system can detect microRNA concentrations ranging from 1 pM to 1 µM with a LOD of 0.12 pM.

**Figure 11:**
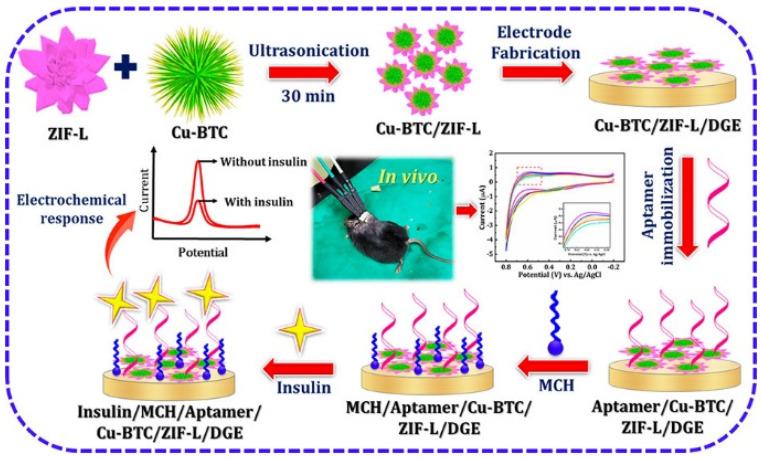
Schematic representation of Cu−BTC/ZIF−L synthesis and its insulin sensing mechanism. Reprinted (adapted) with permission from [96] Copyright {2022} American Chemical Society.

Sakthivel et al. synthesized a composite of two MOFs, such as Cu-BTC nanowire embedded 2D leaf-like ZIF, for the aptamer-based sensor to monitor real-time insulin [96] (Figure 11). Insulin is one of the vital peptide hormones whose primary role is to maintain the normal blood glucose level in the human body. Therefore, fast and sensitive monitoring of insulin levels is very much necessary. To develop the sensor, first, they synthesized CuBTC and Zn-based ZIF-L MOF separately. The morphological studies showed that CuBTC possesses 1D nanowire type morphology while ZIF shows 2D leaf-like morphology with very smooth surfaces. To make the composite, they sonicated CuBTC and ZIF-L in water for 30 min. CuBTC/ZIF-L composite appeared as CuBTC nanowire interconnected 2D leaf-like ZIF-L. To assemble the sensor, the MOF composite was drop cast on disposable Au electrodes (DGE), followed by drop casting insulin aptamer solution onto the modified DGE. Then the mercepto-1-hexanol solution was drop cast to block the non-specific active sites of DGE to minimize the insulin measurement error. Insulin sensing performance was verified by dropping different insulin concentrations on the modified electrode, and the corresponding electrochemical response was measured using differential pulse voltammetry (DPV). This aptamer-based insulin sensor showed linearity within the insulin range of 0.1 pM–5 µM and LOD of 0.027 pM. The sensitivity of the developed aptamer sensor was attributed to the high surface area, enhanced conductivity of 1D/2D MOF, and good electrocatalytic activity. Further, the sensor was grafted in diabetic and non-diabetic mice, and they observed that insulin levels could be efficiently quantified by the developed sensor.

Zhou et al. employed a bimetallic MOF architecture, i.e., Zn MOF on Zr MOF, as an aptamer sensor for sensitive detection of protein tyrosine kinase-7 (PTK7). PTK7 is a vital cancer biomarker that plays a pivotal role in regulating neural development, planar cell polarity in vertebrates, and morphogenetic cell movement during embryonic development [97]. They synthesized Zn MOF using 2-methylimidazole (2-mim) as an organic linker, whereas, for Zr MOF, they used 4′,4′′′,4′′′′′-Nitrilotris [1,1′-biphenyl]-4-carboxylic acid (H_3_NBB) as an organic ligand. To design the aptamer sensor, MOF solution was first dropped cast on the Au electrode, and then the aptamer solution for detecting PTK7 was immobilized. Generally, linkers in MOF possess different functional groups that offer π-π stacking, hydrogen bonding, and electrostatic interaction with negatively charged nucleic acid sequences. This helps in better immobilization of aptamer on the MOF surface. Later PTK7 sensing performance was evaluated by impedance study and differential pulse voltammetry (DPV). They observed that as the aptamer was immobilized on the MOF-modified Au electrode, impedance increased compared to bare Au or MOF-modified Au. This is because aptamer can ionize into negatively charged species, thus repelling negatively charged Fe(CN)^3−^/^4−^ and inhibiting the flow of Fe(CN)^3−^/^4−^ to the electrode surface, thus causing an increment in the charge transfer resistance. Further, in the presence of PTK7 solution, impedance again increased due to the G-quadruplex interaction between PTK7 and aptamer that blocks the path of the redox ions. A similar trend was observed in DPV measurement, and with the increase in PTK7 concentrations, DPV current decreased. With both electrochemical impedance spectroscopy (EIS) and DPV techniques, a linear range of PTK7 within 0.001–1 ng/mL was achieved. However, LOD obtained by the EIS technique was 0.84 pg/mL^,^ whereas from the DPV technique was 0.66 pg mL^−1^. The developed aptasensor was then implemented in human serum samples, and recovery ranging from 96.6–104.6% was achieved.

Sun et al. developed a black phosphorous nanosheet (BPNS) and thionine (TH) doped 2D Cu-MOF for microRNAs [98]. MicroRNAs are essential biomarkers for many disease diagnoses, such as cancer, heart disease, and many neurological diseases. Therefore, the diagnosis of miRNAs is clinically important. They prepared 2D Cu-MOF using PTTBA as the organic ligand, and TH was doped into Cu-MOF. On the other side, BPNS was prepared by sonicating BP crystals in N-methyl pyrrolidone (NMP). BPNS/TH/Cu-MOF was prepared by slowly adding BPNS dispersion solution into TH/Cu-MOF complex. To prepare the aptamer solution, they first dropped cast Nafion solution on GCE, and then TH/Cu-MOF complex solution was dropped. After drying, ferrocene (Fc) coordinated aptamer solution was dropped on TH/Cu-MOF modified GCE. In the presence of miR3123, due to the formation of a particular complex between Fc aptamer and miR3123, Fc concentration decreased, leading to a decrease in I_Fc_. On the other side, current due thionine I_TH_ didn’t change much. Thus, the ratio of I_Fc_/I_TH_ was utilized as a parameter to evaluate the sensing performance. This strategy led to the selective determination of miR3123, and the sensor showed a wide range linearity of 2 pM–2 µM with a LOD of 0.3 pM.

### 4.3. Immunosensor

Immunosensors are generally based on antibody-antigen reaction, which is clinically important for early diagnosis of disease-related proteins. Not only the high surface area and conductivity of 2D MOFs, but post-synthesis modifications of 2D MOFs also help to introduce selective molecular recognition element that helps to enhance both selectivity and sensitivity. Other advantages of 2D MOFs are the reduced thickness (~nm) that expedites the electron and mass transport, exposed metal sites serving as the active catalytic sites and their lateral dimension, which permits efficient immobilization of the molecules, and surface-active reactions. Owing to these attractive properties, 2D MOFs serve as a potential candidate for electrochemical immunosensor.

**Figure 12:**
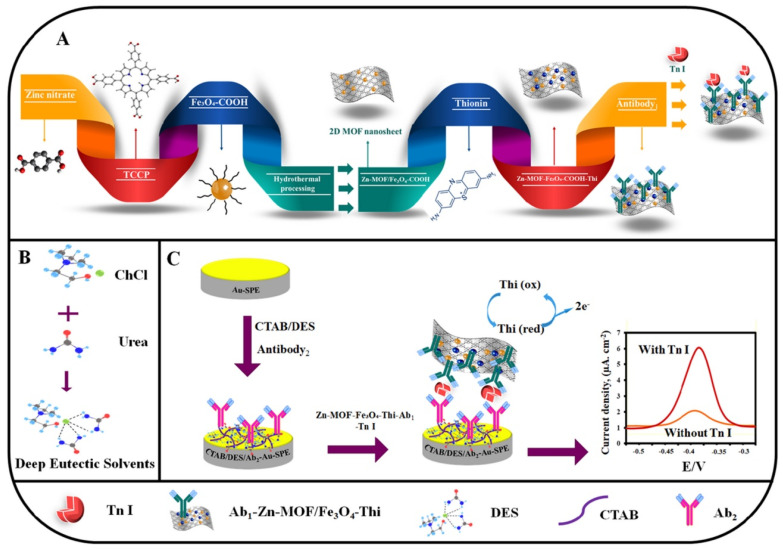
Schematic representation of (**A**) antibody labelled 2D Zn−TCPP MOF/Fe_3_O_4_−COOH/Thi. (**B**) Deep eutectic solvent, (**C**) electrochemical sensing mechanism of CTnI. Reprinted from [99], with permission from Elsevier.

Ahmadi et al. developed an electrochemical immunosensor for detecting cardiac troponin I (CTnI) based on zinc-based 2D Zn-Tetrakis (4-carboxyphenyl) porphyrin (TCPP) MOF functionalized with Fe_3_O_4_-COOH and thionine labeled with anti-CTnI monoclonal antibody (Ab_1_-Zn-MOF/Fe_3_O_4_-COOH/Thi) [99] (Figure 12). CTnI is an important biomarker for the early diagnosis of many cardiovascular diseases, especially acute myocardial infarction (AMI). The level of CTnI in healthy humans usually lies below 0.04 ng/mL, while its level above 0.1 ng/mL indicates an increased risk of heart failure. Therefore, it prioritizes the development of a fast and sensitive sensor for CTnI. In the above-said immunosensor, an Au screen printed electrode (SPE) modified with CTAB/DES (deep eutectic solvent of ChCl, urea) and Ab_2_ (anti-CTnI polyclonal antibody) was used as biosensing surface. The formation of the sensing device was confirmed through the EIS study. Further electrochemical sensing performances were carried out by the DPV technique. DPV response of the developed immunosensor showed an increment in current as the CTnI concentration increased from 0.04 ng/mL to 50 ng/mL. The sensor showed two linear ranges of CTnI: (a) 0.04–0.7 ng/mL and (b)1–50 ng/mL. Consequently, the sensor exhibited a lower LOD and sensitivity of 0.0009 ng/mL and 5.2 µA ng mL^−1^ cm^−2,^ respectively.

**Figure 13:**
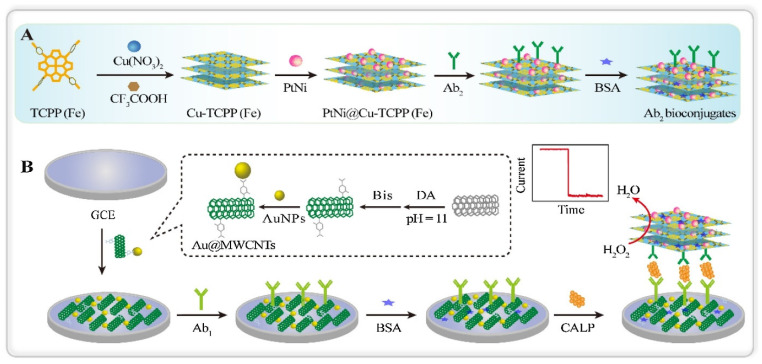
Scheme of (**A**) Fabrication procedure of PtNi@Cu-TCPP(Fe)-Ab_2_ bioconjugates and (**B**) fabrication of electrochemical immunosensor. Reprinted from [100], Copyright (2020), with permission from Elsevier.

Dong et al. prepared a nonenzymatic calprotectin (CALP) immunosensor based on PtNI NPs functionalized Cu-TCPP(Fe) nanosheets [100] (Figure 13). CALP is a calcium-zinc binding protein that is related to the inflammatory response. It is also a serum biomarker for melioidosis disease. In this work, 2D Cu-TCPP(Fe) nanosheets are employed since, due to nanometer thickness, it allows rapid mass transport. Besides, nanosheets also offer excellent catalytic activity and allow high surface active reactions. Additionally, to enhance conductivity further and prevent the accumulation of nanosheets, PtNi nanospheres were immobilized. Here, antibody (Ab_2_) conjugated Ab_2_/PtNi-CuTCPP(Fe) was utilized as a signaling molecule, and Ab_1_ and Au/MWCNT functionalized GCE as the sensing surface. The increasing trend of amperometric current was observed with increasing CALP concentrations that depicted a potential for enzyme-free detection. This is attributed to the excellent electrocatalytic activity of PtNi/Cu-TCPP(Fe) hybrid nanosheets towards the reduction of H_2_O_2_. This developed immunosensor showed a linear range of CALP concentrations within 200 fg/mL–50 ng/mL and a low detection limit of 137.7 fg/mL.

Some of the recent reports on 2D MOF-based electrochemical sensors are summarized in Table 3.

From the above overview, it is understood that 2D MOFs demonstrate appealing properties such as high surface area, flexible porosity, and tunable functionalities, which are advantageous for electrochemical biosensing applications. However, limitations like structural stability and conductivity should be addressed. Structural stability is one of the major concerns for 2D MOF-based sensors. Since electrochemical sensing is done in the aqueous medium, it is essential to design chemically stable 2D MOFs. Therefore, a systematic study on chemical stability with varying functionalities of MOF is required. Further, screening of suitable metal nodes and organic ligands is needed to design chemically stable MOF nanosheets. In this case, theoretical investigations correlating organic ligands and their structural stability in different chemical environments may provide insight into designing such 2D MOF sheets. Although 2D MOFs are more conductive than their bulk counterparts, the conductivity can be further improved by designing redox active MOFs. Another approach to improve conductivity is adjusting the pore aperture. For a conductive 2D MOF, electrocatalytic active centers nearer to each other can accelerate the charge transport through the framework. Therefore, the minimum pore size in the 2D MOF skeleton should be large enough to facilitate the diffusion of ions during electrochemical sensing. At the same time, larger pore sizes are also not always beneficial since they may hinder electron transport across the redox active centers due to increased distance between the active metal sites. Additionally, larger pore sizes will also affect the selectivity of the sensor. Therefore, modulating pore size is necessary to make MOF nanosheets intrinsically conductive and selective towards specific analytes. Alternatively, post-synthesis modification, such as exchanging functional groups, can enhance the stability, conductivity, and surface reactivity of the MOF sheets. As most of the MOF nanosheet-based sensors are employed in the alkaline medium (pH 12), it is necessary to tune the functionalities of 2D MOF for neutral pH (pH 7.4, compatible human serum or blood sample) sensing. Lastly, in-situ spectroscopic techniques (Raman spectroscopy, Fourier transform infrared spectroscopy (FTIR)) need to be performed to understand the MOF-analyte interaction for electrochemical sensing better. 

## 5. 2D MOFs for Optical Biosensors

2D MOFs have also been explored in optical biosensors, although not as extensively as electrochemical biosensors. Optical sensors broadly involve principles of colorimetry, luminescence, surface-enhanced Raman scattering (SERS), and surface plasmon resonance (SPR) [82,86,114]. Detection approaches with and without the use of enzymes can also be found in these sensors. Optical sensors claim to alleviate the drawbacks of electrochemical sensors, such as invasive sample acquisition and signal drifts during in vivo sensing. Colorimetric sensors involve a color change in response to the analyte, whereas luminescence-based sensors exhibit enhancement (turn-on) or quenching of fluorescence in the presence of the analytes [84,115]. For biological analytes, the majority of these sensors utilize MOFs that mimic enzyme action (nanozymes) as these are more stable, cheaper, and suitable in harsh environments as compared to natural enzymes [116]. Such MOFs typically involve porphyrin-based ligands like TCPP(Fe), hemin, etc. [117]. Surface plasmon resonance (SPR) based sensors utilize the change in refractive index occurring due to the interaction of analytes on a metal sensor surface, usually gold [118]. Surface-enhanced Raman scattering (SERS) based sensors involve amplifying the Raman signal of an analyte interacting with the surface plasmon of metals (Au, Ag, etc.) [119]. 

The colorimetric technique is one of the most straightforward optical sensing strategies, which can provide a visual cue of the occurrence of a reaction between a specific agent and the analyte of interest. The color intensity gives a quantitative measure of the concentration of the analyte in the sensing medium. Zeng et al. [84] employed a 2D Cu-TCPP(Fe) based colorimetric sensor for the quantitative detection of proteins. They demonstrated the sensor using a model analyte- carcinoembryonic antigen (CEA), which is useful for the early detection of colorectal cancer. The MOF nanosheets were modified with Au NPs and DNA aptamers to improve their performance. The Au NPs provide binding sites for the DNA and enhances the enzyme-like activity of the MOF, whereas the DNA aptamer specifically binds to the analyte. For colorimetric sensing, they utilized a 96-well plate filled with CEA antibodies. When the CEA protein is added to the well, it gets captured by the antibody. Further, on the addition of the MOF composite, it binds to the target owing to the specific recognition by the aptamer. This leads to the formation of colored products in the presence of tetramethylbenzidene-hydrogen peroxide (TMB-H_2_O_2_) chromogenic substrate, as the MOF has strong horseradish peroxidase (HRP)-like activity. The color change provides a visual cue for the qualitative presence of the protein, whereas the quantitative analysis can be done by measuring the corresponding absorbance wavelength (450 nm). The DNA/Au NP/Cu-TCPP(Fe) sensor showed strong HRP-mimicking activity and excellent selectivity. It exhibited a LOD of 0.742 pg/mL in the linear range of 1 pg/mL to 1000 ng/mL and showed excellent sensing performance in human serum samples.

Luminescence-based sensors offer better sensitivity and lower detection limits than colorimetric techniques and are more popular. These sensors employ the turn-on or -off mechanism (fluorescence enhancement or quenching, respectively) occurring in response to an analyte, with the fluorescence intensity giving a measure of the analyte concentration [115]. The stimuli for the luminescence may be light (fluorescence/photoluminescence), electric current (electroluminescence), chemical reaction (chemiluminescence), an electrochemical reaction (electrochemiluminescence), etc. The fluorescence properties (quenching/enhancement) can be tuned based on the ligand, metal nodes, or guest molecules. Luminescence in MOFs primarily arises from the metal centers (ex.: lanthanides), ligands (ex.: porphyrins), metal-ligand interaction, or additional guest molecules (ex.: fluorescent dyes, quantum dots, lanthanide ions, luminescent complexes) within the MOFs [120]. 

**Figure 14:**
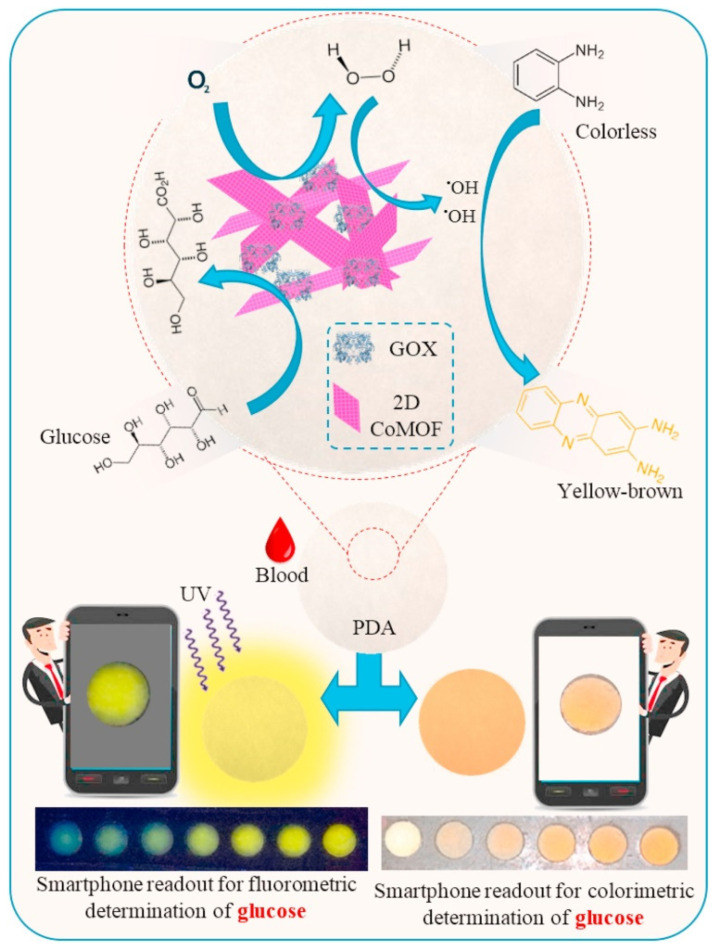
Fluorometric and colorimetric glucose detection by a bi-functional 2D Co-MOF. Reprinted from [121], with permission from Elsevier.

Al Lawati et al. [121] designed a paper-based colorimetric/fluorometric diagnostic device for glucose monitoring by using cobalt-terephthalate MOF (CoMOF) nanosheets acting as the catalyst and enzyme supporting layer (Figure 14). The 2D CoMOF consisted of thin, layered nanosheets of similar thickness, good BET surface area (193.6 m^2^/g), excellent stability (in aqueous solution), and abundant active sites exposed on its surface. The authors utilized the change in color occurring during the reaction between H_2_O_2_ (produced by GOx-assisted oxidation of glucose) and o-phenylenediamine (OPD) to quantify glucose in human blood samples. The oxidized form of OPD (2,3-diaminophenazine) also emitted fluorescence that was proportional to the glucose concentration. The colorimetric and fluorometric signals thus produced were detected by a smartphone-based readout. The sensor exhibited a linear response in the range of 50 µM–15 mM with a LOD of 16.3 µM and 3.2 µM for colorimetric and fluorometric sensing, respectively. The presence of the CoMOF nanosheets served a dual purpose. It catalyzed the peroxide dissociation to hydroxyl radicals as well as provided stable support for the GOx. The GOx exhibited better stability (~60 days) and enhanced glucose oxidation ability (improved reaction speed and sensitivity) in the presence of the MOF, as compared to the presence of horseradish peroxidase (HRP, typically used catalyst). Apart from these, the developed sensor showed a fast response (<1 min), high sensitivity (better than HRP-based systems), high specificity (due to GOx), and excellent real sample recoveries (>95%). In a different example, Qiu et al. [34] developed a multiplex fluorometric sensor for the simultaneous detection of different pathogenic genes by using ultrathin Cu-TCPP nanosheets. The MOF was found to be highly selective towards ssDNA but not dsDNA. This quenches the fluorescence of the labeled DNA. The fluorescence of the label (Texas Red, Cy3, or FAM) is restored when the labeled duplex binds to the target DNA (DNAs of *Salmonella enterica*, *Listeria monocytogenes* and *Vibrio parahemolyticus* used here). Picomolar detection was achieved for the gene segments of all the pathogens. Another interesting work by Liu et al. [81] investigated the effect of pore size and number of layers in 2D cobalt(II)-porphyrin MOF (Co-TTPP MOF, TTPP- 5,10,15,20-tetrakis [4′-(terpyridinyl)phenyl]porphyrin) on sensing DNA by fluorescence method. They precisely controlled the number of layers (1–15 layers) in the MOF by using the Langmuir-Blodgett synthesis technique. They found that highly sensitive DNA detection is achieved for MOFs with an appropriate pore size and an optimum number of layers. At the optimum condition (10 layers), the sensor showed a LOD of 0.1 nM with high selectivity and multiplexing capabilities.

**Figure 15:**
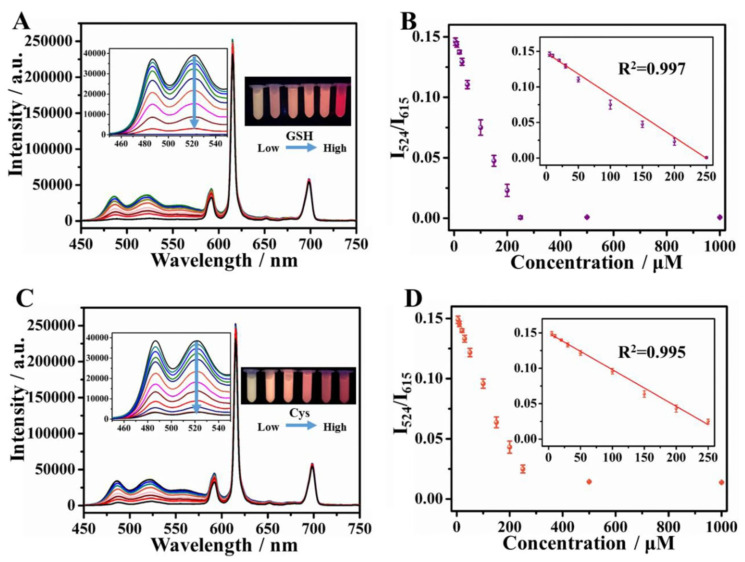
Ratiometric fluorescence sensing of GSH (**A**,**B**) and Cys (**C**,**D**) by Ag/Eu@Ni-MOF nanosheets. (**A**,**C**) represents the emission spectra of the MOFs in response to different concentrations of the analytes (GSH, Cys). Insets show the photos of the MOF in the solutions under UV irradiation (254 nm). (**B**,**D**) indicates the corresponding calibration curves obtained (I_524_/I_615_ vs. concentration of analyte) with the linear fits given in the insets. Reprinted by permission from [Springer Nature Customer Service GmbH]: [Springer Nature] [Nature Materials] [Metal-organic framework nanosheets in polymer composite materials for gas separation, Yun Shu, Tao Dai, Qiuyu Ye, Dangqin Jin, Qin Xu, Xiaoya Hu], [The Author(s), under exclusive license to Springer Science+Business Media, LLC, part of Springer Nature 2021] (2021) [122].

Recently, ratiometric fluorescent sensing has emerged as an effective sensing method as it can evade environmental interferences and manual errors compared to sensing with a single fluorescence intensity. Chen et al. [83] developed a ratiometric fluorescence sensor for the detection of H_2_O_2_ and glucose based on water-soluble MIL-53(Al) MOF nanosheets functionalized with amine groups. These nanosheets exhibited fluorescence at 433 nm, which was quenched with the addition of H_2_O_2_ to the OPD-HRP system. The OPD oxidation also gave rise to a new fluorescence peak at 564 nm. Hence, they utilized the ratio of the intensities of these fluorescence peaks to detect the peroxide and glucose. They observed LODs of 26.49 nM and 0.041 µM in the linear ranges of 0.5–50 µM and 4–42 µM, respectively, for H_2_O_2_ and glucose. Their glucose sensor also exhibited excellent recovery (>97%) in human serum samples. In another work [122], Ni-MOFs were utilized as a stable scaffold for incorporating a luminescent center (Eu^3+^) (Figure 15). The resulting dual-emitting (emission from Eu^3+^ and ligand) MOF was used to fabricate a ratiometric fluorescence-based sensor for detecting cysteine (Cys) and glutathione (GSH). The Eu@MOF was also modified with silver (Ag) which lowers the energy transfer efficiency from ligand to Eu^3+,^ resulting in high ligand fluorescence. In the presence of the analyte, the Ag^+^ ions bind to the –SH groups in them, weakening its influence on the energy transfer process in the MOF. This leads to quenching of the ligand luminescence. The color change was observable by the naked eye. High sensitivity in a wide linear range of 5–250 µM was reported. The LODs obtained were 0.20 µM and 0.17 µM for Cys and GSH, respectively. The sensor also showed good recovery values (92–105%) in human serum samples.

Luminescence sensors employing other excitation events have also been reported. One example is chemiluminescence sensors which involve a chemical reaction that emits light. This method provides ultra-low detection limits with wide detection ranges. Zhu et al. [123] developed a chemiluminescence-based sensor employing cobalt-tetrakis(4-carboxyphenyl)porphyrin (Co-TCPP(Fe)) MOF nanosheets labeled with GOx and luminol for the detection of glucose in human urine. The MOF displayed peroxidase-like activity, which was utilized to catalyze hydrogen peroxide decomposition. The glucose undergoes oxidation in the presence of GOx to form H_2_O_2_, which gets decomposed by the 2D MOF, triggering the oxidation of luminol to produce a chemiluminescence signal. The sensor exhibited glucose detection in the linear range of 32–5500 µg/L with a LOD of 10.667 µg/L, good selectivity, and acceptable recoveries (89–121%) in different samples. Another technique that offers high sensitivity and reduced background signals is electrochemiluminescence (ECL), which utilizes light emitted during an electrochemical reaction to detect analytes. These sensors usually exploit MOFs to provide a platform that can efficiently incorporate suitable ECL reagents without particle aggregation. For instance, Wang et al. [124] reported luminol-Ag NPs loaded Co/Ni-MOF nanosheet microflowers for the detection of alpha-fetoprotein. The MOF nanosheets efficiently incorporated the ECL agent, had high catalytic activity, and resisted the aggregation of the nanoparticles. The sensor exhibited a low LOD of 0.417 pg/mL and good performance in human serum samples. The use of ECL reagents as ligands has also been reported. Yan et al. [114] synthesized nanosheets of a zinc-based MOF (RuMOF) using an ECL luminophore as the organic ligand tris(4,4′-dicarboxylic acid-2,2′-bipyridyl)ruthenium-(II) dichloride (Ru(bpy)_3_^2+^) for ultrasensitive, electrochemiluminescent (ECL) sensing of cardiac troponin I (cTnI). Using Ru(bpy)_3_^2+^ as a ligand instead of as an ECL guest in typical carriers (silica, 3D MOFs, etc.) avoids the problems associated with the leakage of the luminophore and tedious immobilization steps. Additionally, the nanosheet structure improved the ECL efficiency and posed as an excellent matrix for antibody immobilization. The sensor was fabricated employing sandwich-type immunological recognition and exhibited signal-on ECL behavior. It showed excellent sensitivity in a wide linear range of 1 fg/mL–10 ng/mL with a LOD of 0.48 fg/mL. The sensor also showed excellent recovery in human serum samples (95.14–104.75%), good selectivity, and stability for cTnI detection.

**Figure 16:**
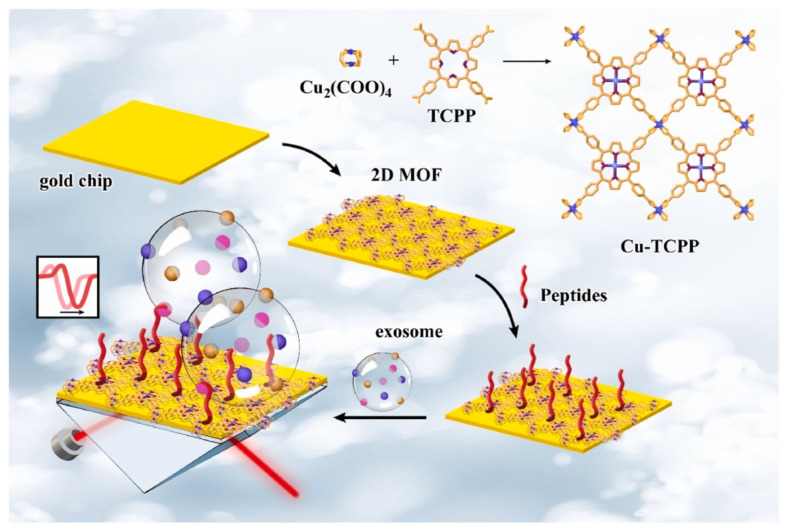
Schematic illustration of a 2D MOF-based surface plasmon resonance biosensor for the detection of PD-L1 exosomes. Reprinted from [125], Copyright (2022), with permission from Elsevier.

Surface plasmon resonance-based sensors are promising for the ultra-sensitive detection of different analytes. Compared to electrochemical, colorimetric, or luminescent sensors, SPR sensors have the advantage of being simple, label-free, and offering real-time assay. Wang et al. [125] developed an SPR-based sensor to detect programmed death ligand-1 (PD-L1) exosomes using a 2D Cu-TCPP MOF-modified gold chip (Figure 16). They observed enhanced performance for the MOF-modified gold chip compared to the bare one (refractive index (RI) sensitivity increased by 39.57°/RIU after modifying with MOF). The 2D MOF improved the excitation electric field and SPR of the sensing interface owing to its high electrical conductivity, charge mobility, and high-efficiency photogenerated carriers. The signal output was enhanced due to the increased incident light absorption, as the MOF has excellent photoelectric properties. The ordered lamellar arrangement of the MOFs also promoted the coupling of its surface plasmon wave with the SPR of the gold. The sensor also exhibited more signal molecule capture. This was due to the combined effect of the high surface area (more active sites for analyte binding) and the porphyrin ligand structure (affinity to carbon-based biomolecules). The proposed sensor achieved a LOD of 16.7 particles/mL and an excellent recovery of 93.43–102.35% in human serum samples.

**Figure 17:**
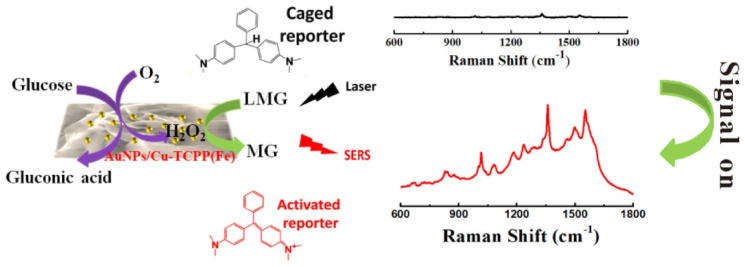
Schematic diagram of a 2D MOF−based, enzyme−free, SERS biosensor for glucose detection. Reprinted (adapted) with permission from [82]. Copyright 2020 American Chemical Society.

SERS-based biosensors utilize the enhancement of Raman responses of analyte molecules on interaction with localized surface plasmons. It offers ultra-sensitive detection of analytes in trace concentrations (up to single molecule detection). It also provides multiplex detection and excellent detection in biological matrixes. Hu et al. [82] employed 2D porphyrinic MOF (Cu-TCPP(Fe)) for a surface-enhanced Raman scattering (SERS) based non-enzymatic glucose assay (Figure 17). They modified the MOF (with peroxidase-like activity) surface with gold nanoparticles, which had GOx mimicking ability. The combined effect of the Au/MOF catalyzed the reaction of glucose to H_2_O_2_ and further the oxidation of leucomalachite green (Raman inactive) to malachite green (Raman active), which produced a Raman signal. A low glucose concentration of 0.16 mM could be detected using this sensor. The selectivity of the sensor was attributed to the gold nanoparticles which exhibited catalysis towards the glucose molecules.

2D MOFs have also been utilized in biosensors based on a photoelectrochemical approach. One of the latest works [85] in this field involves Au NP and aptamer-loaded Yb-TCPP nanosheets to selectively detect SARS-CoV-2. The aptamer bound selectively to the S protein on the virus surface, whereas the MOF and the Au NP were the photoactive components. The Au nanoparticles helped in enhancing the photoactivity of the Yb-TCPP nanosheets owing to the plasmon-induced resonance energy transfer (PIRET) and hot-electron transfer between them. The developed sensor could detect the S protein in a linear range of 0.5–8 µg/mL with a LOD of 72 ng/mL.

Recent literatures employing 2D MOFs for optical biosensors are summarized in Table 4.

In summary, optical sensors employing 2D MOFs exhibit low detection limits and excellent sensitivities in a wide detection range. The ability to tune the optical properties based on the ligand/nodes/guest molecules provides an innovative platform for developing sensors tailored for specific analytes. The high surface area boosts the sensitivity, whereas the specific pore sizes enhance the selectivity toward analytes. The ability of some MOFs to act as nanozymes enables the development of non-enzymatic sensors, which eradicates the drawbacks associated with the use of enzymes in sensors. Optical sensors are non-invasive and hence applicable for in vivo applications. However, elaborate studies on the biocompatibility of 2D MOFs are required before practical use.

## 6. Conclusions and Future Perspectives

In conclusion, this review provides an extensive overview of the synthesis and properties of 2D MOFs and their applications in optical and electrochemical biosensors. The first section of the review deals with the unique properties of 2D MOFs compared to their bulk counterparts, such as conductivity, surface area, flexibility, mechanical strength, etc. Later, the different synthesis techniques of 2D MOF, such as top-down and bottom-up methods, have been discussed briefly. The top-down synthesis approach deals with the delamination of layers from the 3D MOF through different physical or chemical methods. On the other side, the bottom-up approach involves the growth of MOF from a metal source and organic ligand in a chemical approach. Although the top-down approach has various advantages, it also suffers drawbacks concerning yield and stability. The bottom-up approach offers more scope for controllable growth of MOF nanosheets with tunable morphology, porosity, and surface by tuning synthesis parameters and precursor compositions. Therefore, a wise choice of the synthesis method is essential for successfully developing 2D MOFs.

This review also summarizes the 2D MOFs implemented in optical and electrochemical biosensors. MOF nanosheets possess exciting properties such as high surface area, which provides abundant sites for biomolecule adsorption that is beneficial for both optical and electrochemical sensors. 2D MOF-based optical sensors are based on different optical phenomena such as luminescence, SPR, fluorescence, etc., discussed in this review. High surface area is also advantageous for enhancing mass transfer and thus contributes to the enhanced catalytic activity of the 2D MOF-based electrochemical sensors. Besides, specific pore dimensions can easily be tuned in MOF sheets which further improves the selectivity of the electrochemical sensor. The role of 2D MOF in electrochemical sensors such as bio-analyte (glucose, DA, UA, H_2_O_2_, etc.) sensors, nucleic acid sensors, and immunosensors are reviewed in this work.

**Figure 18:**
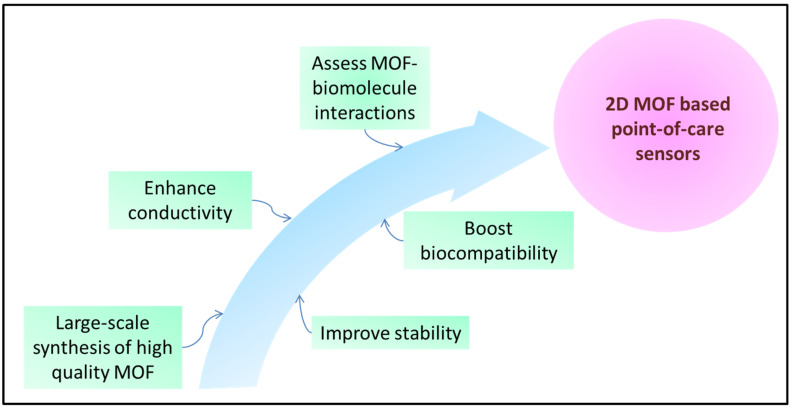
Future perspectives for the development of 2D MOFs as point-of-care sensors.

Although 2D MOFs have unique properties to be applied as biosensors, a few challenges must be addressed for their widespread implementations (Figure 18).

- One of the major issues with MOFs is their stability in different solution media. Metal centers and organic linkers in MOF nanosheets are very sensitive to the pH as well as the functional groups present in the analyte solution.
- Although 2D MOFs have shown better conductivity than 3D MOFs, they still suffer from poor conductivity when utilized for sensing without any modifications.
- Despite several reports, the growth mechanism of 2D MOFs is still poorly understood. Apart from this role of MOF sheets in biosensing applications and the interaction between MOF and biomolecules are not well established.

Despite the above-mentioned challenges and due to the attractive properties of 2D MOFs, they are proposed to be potential candidates for optical and electrochemical biosensing applications. Optical and electrochemical detection of bioanalytes using MOF sheets are still in the infant stage and requires extensive research to investigate the role of MOFs. Suitable synthesis methods with high yield, good stability, and high-quality MOF sheets need to be probed and developed in the future. From the sensing perspective, MOF-biomolecule interaction needs to be thoroughly studied and investigated for the practical implementation of 2D-MOFs in optical and electrochemical biosensors.

## Figures and Tables

**Table 1:** Comparison of 2D MOFs with other 2D nanomaterials.

2D Material	Structure	Surface Area	Electrical Conductivity	Thermal Conductivity	Stability	Other Properties	Ref.
Graphene	sp^2^ carbon arranged in a hexagonal, honeycomb lattice	High (~2630 m^2^/g)	Very High	High (~4000 Wm^−1^K^−1^)	Stable in most conditions	Excellent mechanical properties (~130 GPa fracture strength). Excellent optical properties.	[2]
Graphene oxide	Graphene structure rich with oxygen functional groups	Smaller than graphene	Insulating/semiconducting	High; lower than graphene	Stable in water	Bulk synthesis possible. Easily modifiable surface owing to functional groups.	[2]
TMD	MX_2_; a layer of transition metal atoms (M) sandwiched between two layers of chalcogen (X) atoms	Low (~16 m^2^/g for MoS_2_ nanosheets)	Metallic to semiconducting depending on phase	2–3 orders lower than graphene	Depends on synthesis and modification	Excellent for optoelectronics.	[5,9]
MXene	Transition metal atoms (M) arranged in a hexagonal structure, with the octahedral sites filled by carbon or nitrogen (X)	Moderate	High	Moderate	Sensitive to water and oxygen	Excellent mechanical properties. Interesting optical and electrochemical properties. EMI shielding property.	[10]
LDH	Brucite-like cationic layers with intercalated anions for charge neutralization	Moderate	Low	Low	Dependent on type of component ions	Easy synthesis. Excellent ion exchange capacity, ionic conductivity.	[4]
MOF	Metal ions or clusters linked by organic ligands	Very high (~1000–10,000 m^2^/g)	Low; higher than 3D MOF	Low	Depends on type of MOF; more stable than 3D MOF	In-situ and post-synthetic modification possible. Tunable pore size. High porosity. High mechanical strength and flexibility.	[8]

TMD—Transition metal dichalcogenides. LDH—Layered double hydroxides.

**Table 2:** Comparison of the nonenzymatic glucose sensing properties of 2D MOFs with other materials.

Catalyst	Medium	Sensitivity (µA mM^−1^cm^−2^)	Linearity (mM)	Limit of Detection (µM)	Stability/Real Sample (Recovery)	References
Amorphous Co-Ni hydroxide	0.5 NaOH	1911.5	0.00025–5	0.12	60 days, 102%	[21]
CNF/Co(OH)_2_	0.1 M NaOH	68,000	0.01–0.12	5	-	[22]
Pt@CNO	PBS (pH 7.4)	21.6	2–28	90	-	[23]
Ni(TPA)MOF-SWCNT	0.1 M KOH	-	0.02–4.4	4.6	15 days, human serum	[24]
Pt-Pd/PHNG	0.1 M PBS (pH 7)	52.53	0.1–4	1.82	3 weeks, human blood (101.43%)	[25]
Hierarchical sheet-like Ni-BDC/GCE	0.1 M NaOH	636	0.01–0.8	6.68	-	[26]
Co-MOF nanosheet array/NF	0.1 M NaOH	10,886	0.001–3	0.0013	7 days, Fruit juice, human serum	[27]
Ni-MOF@Ni-HHTP-5	0.1 M NaOH	2124.90	0.5–2665.5	0.02	-	[28]

CNF: Carbon nano fibre. CNO: Carbon nano-onion. SWCNT: Single-walled carbon nanotube. NF: Nickel foam. PHNG: Porous holey nitrogen doped graphene. HHTP: 2,3,6,7,10,11-hexahydroxytriphenylene.

**Table 3:** Summary of 2D-MOF based electrochemical biosensors.

Sensor	Method	Analyte	Linear Range (µM)	Sensitivity (µA mM^−1^ cm^−2^)	LOD	Real Sample/Recovery	Ref.
Ultrathin Ni MOF	CA	Glucose	25–3160	402.3	0.6 µM	Human serum (97–104.7%)	[101]
Vertical 2D NiCo MOF nanosheet	CA	Glucose	1–8000	684.4	0.29 µM	Human serum (96–106%)	[102]
Ni-MOF@HHTP	CV and CA	Glucose	0.5–2665.5	2124.90	0.0485	-	[28]
2D/3D NiCu MOF-6	CA	Glucose	0.02–4.93	1832	15	Human serum (94.5–97.3%)	[103]
Co-MOF nanosheet array/Ni foam	CV and CA	Glucose	1–3000	10,886	1.3 nM	Blood serum, fruit juice	[27]
Ni@Cu-MOF nanosheet	CV	Glucose	5–2500	1703.33	1.67	Human serum (100–104%)	[104]
2D MOF-74 (Cu) nanosheet	CA	Glucose	100–1000	3810	0.41	Human serum	[105]
NH_2_-GP/2D arrays of Cu_3_(btc)_2_	CV and CA	Glucose	0.05–1775.5	5360	30 nM	Human sweat sample	[93]
NH_2_-GP/2D arrays of Cu_3_(btc)_2_	CV and CA	Lactate	0.05–22.6 mM	29	5	Human sweat sample	[93]
2D Cu-TCPP/MWCNT	CA	H_2_O_2_	1–8159	157	0.70	Human serum (104.7%) Beer sample (103.4%)	[106]
2D Co-MOF@Nafion	CV and CA	H_2_O_2_	5–1000 10^3^–10^5^	570 ± 5 A mM^−1^cm^−2^ 395 ± 10 A mM^−1^cm^−2^	-	Commercial lens cleaning (103%) and commercial disinfectant solution (97%)	[91]
2D Ni-MOF/Hemin	CV and DPV	H_2_O_2_	1–400	38	0.2	Human serum (99.32–101.88%) Disinfectant water sample	[107]
Co-MOF	CA	H_2_O_2_	0.5–832.5	0.0412 (µA µM^−1^)	0.47	-	[108]
Ni-MOF	CA	H_2_O_2_	1–3300	0.041 (µA µM^−1^)	1.58	-	[108]
NiCo-MOF	CA	H_2_O_2_	1–830	0.045 (µA µM^−1^)	1.07	-	[108]
Au@Cu_2_O-MIL 53(Fe)	CA	H_2_O_2_	10–1520	351.57	1.01	A549 cells	[90]
Ni-MOF	CA	Ascorbic Acid	0.5–8065.5	2.4	0.25	-	[109]
Co-MOF	CV and CA	Urea	500–7500	5	414	-	[94]
NiCo-MOF	CV and CA	Urea	0.5–332.5	860	6.188	Milk sample	[94]
Ni-MOF	CV and CA	Urea	0.5–832.5	1960	0.471	Milk sample	[94]
{100} facets of Ni_3_(HHTP)_2_	CV	Dopamine	-	-	9.9 ± 2 nM (PBS) 214 ± 48 nM (CSF)	-	[89]
2D/2D NiCo-MOF/Ti_3_C_2_	DPV	Acetaminophen	0.01–400	0.043	0.008	Serum (98.9%) and urine (99.8%)	[92]
2D/2D NiCo-MOF/Ti_3_C_2_	DPV	Dopamine	0.01–300	0.1	0.004	Serum (102.2%) and urine (98.3%)	[92]
2D/2D NiCo-MOF/Ti_3_C_2_	DPV	Uric acid	0.01–350	0.052	0.006	Serum (100.6%) and urine (98.3%)	[92]
FC labeled ssDNA aptamer/BPNSs/TH/Cu-MOF	SWV	microRNA (miR3123)	2 pM–2 µM	-	0.3 pM	Human serum (97.68–104.4%)	[98]
Cu-BTC/ZIF-L	DPV	Insulin	0.1 pM–5 µM	-	0.027 pM	In vivo animal	[96]
2D Zn MOF on Zr MOF	EIS, DPV	PTK7	0.001–1 ng/mL	-	0.84 pg/mL (EIS) 0.66 pg/mL (DPV)	Human serum (96.6–104.6%)	[97]
Co-MOF@TPN-COF	EIS	Ampicilin	0.001–2000 pg/mL	-	0.217 × 10^−3^ pg/mL)	Human serum (95.5–99.9%) River water (98.2–103.4%) Milk (96.4–102.6%)	[110]
Co-MOF/ITO (flow homogeneous assay)	CV and DPV	MicroRNA	1 pM-1 µM	-	0.12 pM	Human serum (98.7–109%)	[95]
2D Zr-MOF (521-MOF)	EIS	Mucin 1 (MUC 1)	0.001–0.5 ng/mL	-	0.12 pg/mL	Human serum (94.8–106.8%)	[111]
Ab_1_-Zn-MOF/Fe_3_O_4_-COOH/Thi signal molecule and Ab_2_/pCTAB/DES as biosensing device	DPV	Cardiac troponin (CTnI)	0.04 ng/mL–50 ng/mL	-	0.0009 ng/mL	Whole blood sample	[99]
PtNi@Cu-TCPP(Fe)	CA	Calprotectin (CALP)	200 fg/mL–50 ng/mL	-	137.7 fg/mL	Human Serum (94–100.9%)	[100]
Anti_NSE_/Zr-TAPP	EIS and DPV	Neuron specific enolase (NSE)	10.0 fg/mL–2.0 ng/mL	-	7.1 fg/mL	Human serum (93.3–106.9%)	[112]
GO@Ab_2_/Ab_1_/BSA/Ag/Cu-TCPP(Fe)/MWCNT	CA	Sulfonamide	1.186–28.051 ng/mL	-	0.395 ng/mL	Water samples (64–118%)	[113]

HHTP: 2,3,6,7,10,11-hexahydroxytriphenylene. TCPP: Tetrakis (4-carboxyphenyl) porphyrin. Fc:Ferrocene. BPNS: Black phosphorous nanosheet. TPN: terephthalonitrile. COF: Covalent organic framework. pCTAB: Polymer film of cetyltrimethylammonium bromide. DES: Deep eutectic solvent. TAPP: 5,10,15,20-tetra(4-aminophenyl) porphyrin. Ab_2_: Polyclonal antibody. Ab_1_: Monoclonal antibody. BSA:Bovine serum albumin.

**Table 4:** Summary of 2D-MOF based optical biosensors.

2D MOF	Analyte	Method	Linear Range	LOD	Real Sample Test in	Remarks	Ref.
DNA/Au NP/Cu-TCPP(Fe)	Carcinoembryonic antigen	Colorimetric	1 pg/mL to 1000 ng/mL	0.742 pg/mL	Human serum	MOF has HRP-like activity	[84]
BODIPY@Eu-MOF	F^−^	Ratiometric fluorescence	0–30 µM	0.1737 µM	Living cells	Low cytotoxicity. Also used for bioimaging	[126]
	H_2_O_2_		0–6 µM	6.22 nM			
	Glucose		0–6 µM	6.92 nM			
Cu@Cu-FeTCPP	Glucose	Colorimetric	0.05–1.25 mM	12 µM	-	High peroxidase mimicking activity	[45]
Cu-TCPP/Au chip	PD-L1 exosome	SPR	10^4^–5 × 10^6^ particles/mL	16.7 particles/mL	Human serum	Higher RI sensitivity (137.67°/RIU), detection accuracy (0.77), and quality factor (24.81 RIU^−1^) were enhanced compared to bare gold sensor	[126]
NH_2_- MIL-53(Al)	H_2_O_2_	Ratiometric fluorescence	0.5–50 µM	26.49 nM	Human serum	NH_2_ groups improve the water-stability of the MOF	[83]
	glucose			0.041 µM			
Ag/Eu@Ni-MOF	GSH	Ratiometric fluorescence	5–250 µM	0.17 µM	Human serum	-	[122]
	Cysteine			0.2 µM			
Au NP/Cu-TCPP(Fe)	Glucose	SERS	0.16–8 mM	0.16 mM	Human saliva	Au: GOx-like activity 2D MOF: peroxidase-like activity	[82]
Co-BDC	Glucose	Colorimetric	50 µM–15 mM	16.3 µM	Human blood	-	[121]
Eu@BCP	Anthrax biomarker	Fluorescence	0–35 µM	0.038 nM	-	Dual-emission	[86]
Tb@BCP				0.033 nM			
Luminol-AgNPs@Co/Ni-MOF	Alpha-fetoprotien	Electrochemiluminescence	1 pg/mL–100 ng/mL	0.417 pg/mL	HUman plasma	Enhanced ECL performance	[124]
Zn-Ru(dcbpy)_3_^2+^	Cardiac troponin I	Electrochemiluminescence	1 fg/mL–10 ng/mL	0.48 fg/mL	Human serum	ECL luminophore utilized as the organic ligand	[114]
Co-TCPP(Fe)	Glucose	Chemiluminescence	32–5500 µg/L	10.667 µg/L	Human urine	Peroxidase-like catalysis	[123]
Co-TTPP	DNA	Fluorescence	0–1 nM	120 pM	-	Best performance for MOF with 10 layers	[81]
In-aip	H_2_O_2_	Fluorescence	0–160 µM	0.87 µM	Human serum	Enzyme assisted analysis. 2D morphology facilitates efficient capture of unstable intermediates and ensures stable luminescence	[127]
	Glucose		0–200 µM	1.3 µM			
Cu-TCPP	*Salmonella enterica* DNA	Fluorescence	0.5–15 nM	28 pM	-	Multiplex detection	[34]
	*Listeria monocytogenes* DNA		0.1–12 nM	35 pM			
	*Vibrio parahemolyticus* DNA		0.1–9 nM	15 pM			
Ni-MOF	H_2_O_2_	Colorimetric	0.04–160 µM	0.008 µM	Human serum	MOF exhibited greater affinity towards TMB and H_2_O_2_ than HRP	[116]
ZIF67	H_2_O_2_	Colorimetric	100–1000 mM	0.11 mM	-	Catalytic activity dependent on pH and reaction temperature	[44]

DNA—deoxyribonucleic acid. TCPP(Fe)—Fe(III) tetra(4-carboxyphenyl)porphyrin chloride. BODIPY—Boron-dipyrromethene. H_2_O_2_—hydrogen peroxide. PD-L1—programmed death ligand-1. BCP—Barium-dipicolinic acid coordination polymer. Ru(dcbpy)_3_^2+^—tris(4,4′-dicarboxylic acid-2,2′-bipyridyl) ruthenium(II). TTPP—5,10,15,20-tetrakis [40-(terpyridinyl)phenyl]porphyrin. ZIF—Zeolitic imidazolate framework. Aip—aminoisophthalic acid. HRP—horseradish peroxidase. TMB—3,3,5,5-tetramethylbenzidine.

## Data Availability

No new data were created or analysed in this study. Data sharing is not applicable to this article.
